# Capability, Opportunity, Motivation, and Behaviour (COM-B) model association with Egyptian dentists’ reporting of suspected abuse

**DOI:** 10.1186/s12903-022-02094-5

**Published:** 2022-03-04

**Authors:** Maha El Tantawi, Nouran Nabil, Sawsan H. Mahmoud, Fatma Elhendawy

**Affiliations:** 1 Department of Pediatric Dentistry and Dental Public Health, Faculty of Dentistry, Alexandria University, Champolion St., Azarita, 21521 Alexandria Egypt; 2 Department of Pediatric, Oral Health and Preventive Dentistry, Faculty of Dentistry, Tanta University, Tanta, Egypt

**Keywords:** Egypt, Mandatory reporting, Abuse, Dentists, Hotlines, Behaviour

## Abstract

**Background and objectives:**

This study assessed the frequency of reporting suspected abuse by Egyptian dentists who have examined patients with manifestations of abuse and factors associated with this reporting within the framework of the Capability, Opportunity, Motivation, and Behaviour (COM-B) model.

**Methods:**

A cross-sectional study included dentists practicing in Egypt in 2019. A questionnaire collected information about personal and professional background, and whether: participants received training to manage abuse, reported suspected abuse, were aware of the presence of hotlines for reporting and agencies supporting abuse victims, and eight items assessing attitude towards reporting suspected abuse. Principal Component Analysis (PCA) was used to assess the structure of attitude items. Logistic regression assessed the relationship between the dependent variable (reporting suspected abuse) and independent factors: receiving training (capability), attitude components (motivation), and awareness of the presence of hotlines and support agencies (opportunity).

**Results:**

The response rate was 68.2% (821/ 1203), mean age (SD) = 29.7 (10.0) years, 43.1% had examined patients with suspected abuse last year and 4.3% reported their suspicions. PCA identified two attitude components scored out of 10: professional attitude towards reporting (mean (SD) = 6.7 (2.2)) and negative perception of workplace commitment to reporting (mean (SD) = 7.2 (2.1)). Higher odds of reporting suspected abuse were associated with better professional attitude towards reporting (AOR = 1.87, P = 0.003) and less negative perception of workplace commitment to reporting (AOR = 0.77, P = 0.04), but not with previous training (P = 0.74), awareness of the presence of victims’ support agencies (P = 0.68) or a hotline (P = 0.88).

**Conclusions:**

Only a minority of dentists reported suspected abuse. Dentists who reported their suspicions had better professional attitude towards reporting and better perception of their workplace commitment to reporting. Thus, the motivation component of the COM-B framework was significantly associated with reporting suspected abuse. The present training methods to manage abuse, and dentists’ unawareness of national efforts to manage the problem do not seem to encourage reporting.

## Background

Abuse is physical, sexual, emotional, economic, or psychological actions or threats of actions that influence another person resulting in injury, psychological harm, deprivation, or death [1]. It includes child maltreatment, violence against women, and elder abuse. Despite the efforts made for preventing abuse by establishing legislation and service programs [2], much remains to be done. Global statistics indicate that most cases of violence against children, women, and the elderly are not reported to authorities [3]. Some possible factors that hinder the decision to report suspected abuse include concern that reporting will not help the family or would damage the relationship with the family, lack of knowledge about the signs of abuse, and not knowing where to report [4]. This highlights the need for reporting suspected abuse. Mandated reporting of suspected abuse and protecting health care professionals to carry this duty empower them to help those who cannot protect themselves [5].

Dentists have a frequent contact with patients compared to other healthcare professionals since people usually visit dentists at least once a year [6]. Moreover, many surveys have shown that 50–77% of abuse cases involve the head and neck region manifested as bruising, abrasions, or lacerations of oral cavity structures; dental fractures, dental dislocations, dental avulsions; maxilla and mandible fractures [7], thus placing dentists in a strategic position to detect, diagnose, document, and report suspected abuse to the authorities [8] to maintain the wellbeing of their patients. Most dentists indicate their readiness to report suspected abuse although lack of clear instructions about the reporting procedure may reduce the chances of translating this intention to actual reporting behaviour [9]. Despite their willingness to report suspected abuse, dentists may be less likely to report than other healthcare professionals [10].

Due to cultural differences, what would be labelled as abuse in Western countries, may not be culturally considered as intentional harm requiring reporting in Arab countries [11]. In Arab societies, some population subgroups may view certain forms of abuse as a private, personal, or family problem rather than a social and criminal problem requiring intervention. Some incorrectly believe that abuse is a justifiable response to misbehaviour [12]. This could be an underlining reason for not reporting such cases. In Egypt, the proportion of women experiencing violence at least once in their lifetime was reported to reach 26% [3]. In addition, 93% of children aged 1–14 years old have been exposed to violent disciplinary practices [13]. Abuse, of any kind or form, cannot be condoned or accepted and must be eradicated. In 2009, the Egyptian government set a national strategy to combat all forms of abuse. This encompassed activating laws, setting up hotlines to report suspected abuse, raising awareness, and establishing agencies concerned with supporting victims and helping them to recover [14]. Furthermore, the National Council for Women was established to take care of issues related to the protection of children and mothers in Egypt. It also supports women subjected to threats of violence and abuse [15].

A previous study assessed dentists’ knowledge, attitudes, and ability to recognize and report suspected abuse in Egypt and showed that 45.1% of participants have suspected child abuse within the previous year and 19.2% reported their suspicions [16]. However, this study, conducted a decade ago, included 182 participants and only 123 of them (67.6%) were dentists and the remaining participants were dental students and interns. In addition, another study [17] reported that Egyptian dentists had one of the lowest levels of intended reporting of suspected abuse among dentists from eight Arab countries. Understanding the level of reporting suspected abuse has implications for education and professional development activities. It is also important to understand the factors that may explain this professional decision to report and their relationship with the COM-B model. This model of behaviour proposes that to engage in a behaviour (B), a person must have physical and psychological capabilities (C) and opportunities (O), and the motivation to demonstrate the behaviour (M) [18]. The COM-B model of behaviour is widely used to identify what needs to change in order for a behaviour change intervention to be effective [19]. Reporting suspected abuse is a desired behaviour that needs to be promoted and the COM-B framework provides actionable insights that can be used to develop interventions for this promotion. The present study assessed the frequency of reporting by dentists who have examined patients suspected of being abused and factors associated with this reporting within the framework of the COM-B model. The null hypothesis was that dentists’ reporting of suspected abuse would not be associated with the COM-B framework constructs.

## Methods

A cross-sectional study was conducted in Egypt and data were collected from April to December 2019 after obtaining the approval of the research ethics committee in the Faculty of Dentistry, Alexandria University and in accordance with the guidelines of the Declaration of Helsinki.

The sample size was calculated [20] assuming 95% confidence level and 5% margin of error with estimated percentage of dentists reporting suspected abuse = 19% [16]. The number was increased by 20% to allow for non-response and a minimum sample size of 283 dentists was needed. Sample size was also planned to accommodate the principal component analysis, ranging from 20 observations per item [21] with a total of 160 for the 8 items used in the PCA to a maximum of 500 as reported in the literature [22].Also, for the logistic regression, at least 500 would be needed [23].

Dentists were recruited using convenience sampling from the network of collaborators/ data collectors in five large dental schools to ensure geographic representativeness all over Egypt: two in the greater Cairo area, one in Alexandria, one in the Delta region, and one in Southern Egypt. Dentists were also recruited from members of the Egyptian Dental Syndicate and from those attending the two biggest conferences in Egypt sponsored by Cairo University and Alexandria University. Dentists were invited to participate if they (1) had a bachelor of dentistry degree and (2) were practising in Egypt during the study period. Paper-based copies of the questionnaires were distributed to dentists in the network of the academics in the 5 dental schools by mail or a courier and were also distributed through the organising committees of the two conferences. In addition, copies of the questionnaire were left at the offices of the Syndicate in different cities where receptionists presented them to visiting dentists. No other dental care personnel were included because the Egyptian healthcare system does not include dental hygienists, or nurses.

Data were collected using a questionnaire that was developed by the first author in a previous study based on the literature [17] and questions that were not relevant to the present study aim were removed. The questionnaire consisted of three sections including close-ended questions. The first section sought information about personal and professional backgrounds: age in years, gender (male or female), type of practice (private, public or academic sectors), receiving training to manage victims of abuse (yes or no), and number of patients examined in the previous year with suspected exposure to abuse. The variable “years of practice” was not included because it would be collinear with age since in Egypt, students are admitted to dental school directly after high school when they are 19 years old. The second section assessed the attitude towards reporting suspected abuse. Participants were asked to indicate how much they agreed with each of eight statements on a scale from completely disagree (score 1) to completely agree (score 10). Two items were reverse coded so that they would indicate positive professional attitude similar to the other items: too busy treating patients not to report and thinking that abuse is a family issue. In the third section, respondents were asked whether they reported suspected abuse after examining a patient. There was a list to check to indicate whom the dentists reported to including the police, Ministry of Social Affairs, Ministry of Health, non-governmental organisations (NGOs), or others. This section also assessed whether the participant was aware of the presence of governmental agencies in Egypt providing support to abuse victims and a hotline for reporting suspected abuse.

The questionnaire was pilot tested in Egypt on ten dentists to ensure clarity of terms and that it covered the studied constructs and had good content validity index (CVI). The calculated CVI was 0.89 indicating good validity. The responses of these ten dentists were not included in the final analysis. The questionnaire was preceded by a brief overview of the study purpose and explained that by responding, the dentist indicated consent to join the study. Thus, informed consent was obtained from the dentists participating in the study. The questionnaire took about 10 min to complete and was self-administered in English.

**Fig. 1:**
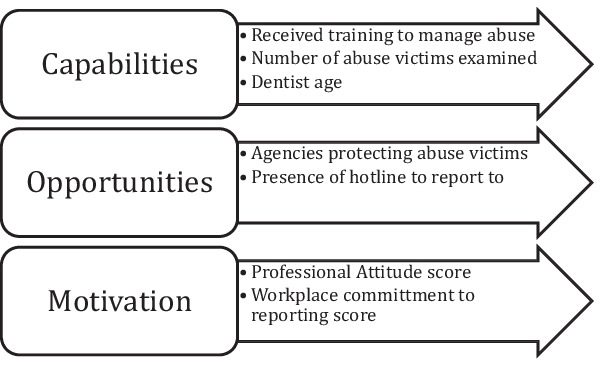
COM-B model explaining reporting suspected abuse

Descriptive statistics were calculated as frequencies and percentages for categorical variables and means and standard deviations or medians and interquartile ranges for quantitative variables. The whole sample and the group of dentists indicating that they had examined patients with suspected abuse in the previous year were compared using t-test and chi-square test. Principal component analysis (PCA) was used to assess the structure and components in the eight attitude statements and Kaiser–Meyer–Olkin (KMO) test was calculated along with the p-value of Bartlett’s test to confirm the suitability of the data for PCA. Scree plot was used to visualize the components with eigenvalues ≥ 1 and based on this, two components were identified. The internal consistency of the items in the two components identified by PCA was assessed using Cronbach alpha. The scores of these two components were calculated by averaging the scores of the items forming each component. A logistic regression model was developed to assess factors associated with the dependent variable: reporting suspected abuse (yes/ no) and the constructs of the COM-B framework (Fig. 1) where capabilities were measured by whether the dentist received training to manage abuse victims, the number of suspected abuse victims the dentist examined indicating repeated exposure to these patients leading to accumulation of experience and dentist’s age also as an indicator of dentist’s experience; opportunities were measured by dentist’s awareness of the presence in Egypt of agencies supporting victims of abuse and the presence of a hotline to report suspected abuse which indicate a general governmental system supportive of reporting suspected abuse and; motivation represented by the two components of the attitude items.

## Results

The response rate was 68.2% (821/ 1203). Table 1 shows that 354 out of 821 dentists (43.1%) indicated that they examined patients with suspected abuse during the past year. Most participants in the whole sample and in the group encountering patients with suspected abuse in the previous year were females (60.4% and 56.7%) with no training in managing abuse (77.4% and 60.2%), and on average, they had examined 8.5 and 14.5 patients suspected of being abused (median = 0 and 3.5, interquartile range = 5 and 19).Dentists who indicated that they had examined patients with suspected abuse were significantly older, more likely to be working in the public sector, have received training to manage abuse victims, and have seen more patients with suspected abuse (P < 0.05). Only 4.3% of the dentists, who indicated they had examined patients suspected of being abused, reported their suspicions.

**Table 1:** Profile of dentists in the whole sample and those indicating they have examined suspected cases of abuse in the previous year

Factors	N (%)	
	All dentists N = 821	Dentists examining patients suspected of being abused last year N = 354
Gender		
Male	323 (39.6)	153 (43.3)
Female	492 (60.4)	200 (56.7)
Age		
Mean (SD)	29.7 (10.0)	31.5 (9.0)
Type of practice		
Public sector	443 (55.8)	221 (63.5)
Private	174 (21.9)	71 (20.4)
University	177 (22.3)	56 (16.1)
Received training to manage abuse		
Yes	185 (22.6)	141 (39.8)
No	634 (77.4)	213 (60.2)
Number of patients suspected of being abused examined		
Mean (SD)	8.5 (29.1)	14.5 (36.7)
Aware of governmental agencies protecting abuse victims		
Yes	112 (13.6)	62 (17.5)
No/not sure	709 (86.4)	292 (82.5)
Aware that there is a hotline to report suspected abuse		
Yes	63 (7.7)	31 (8.8)
No/not sure	758 (92.3)	323 (91.2)
Reported when abuse was suspected		
Yes	–	15 (4.3)
No	–	337 (95.7)

Table 2 shows the KMO = 0.72 and P-value of Bartlett’s test < 0.0001 indicating the suitability of data for PCA. PCA identified two components: component 1 including 5 items assessing professional attitude towards reporting suspected abuse, Cronbach alpha = 0.66. The second component included three items assessing participants’ negative perception of workplace commitment to reporting suspected abuse with alpha = 0.58. The items in the two components generally had strong factor loadings. The mean (SD) score of the five items in the first component was 6.7 (2.2) out of a maximum score of 10 and of the three items in the second component was 7.2 (2.1) out of a possible maximum of 10.

**Table 2:** Factor loadings for two components of attitude towards dentists’ reporting of suspected abuse

Items	Mean (SD)	Factor loadings	
		Component 1 Professional attitude towards reporting	Component 2 Negative perception of workplace commitment to reporting
Not enforced	7.4 (2.6)		0.703
Not mandated at workplace	6.6 (3.0)		0.693
No designated authority to report to	7.7 (2.7)		0.751
Right thing to do	8.0 (2.9)	0.568	
Never too busy treating patients not to report	6.5 (3.2)	0.729	
Not think abuse is a family issue	6.9 (3.0)	0.749	
Mandated by law	6.0 (3.3)	0.472	
Considers part of the job	6.2 (3.3)	0.651	

KMO = 0.72, P of Bartlett’s test < 0.0001, 2 components explained 48.6% of variance

Alpha of items in component 1 = 0.66, mean (SD) of 5 items = 6.7 (2.2) out of max of 10

Alpha of items in component 2 = 0.58, mean (SD) of 3 items = 7.2 (2.1) out of max of 10

The greatest percentage of dentists indicated that they would report cases of suspected abuse to Ministry of Health (31.9%), the police (27.7%), and Ministry of Social Affairs (13.6%). A minor portion indicated that they would report to NGOs (5.4%) and other bodies (1.7%) while 22.3% did not know who to report to.

Table 3 shows that in logistic regression there were not statistically significant associations between reporting suspected abuse and receiving training to manage abuse victims (P = 0.74), examining greater number of patients suspected of being abused (P = 0.52), older age of dentist (P = 0.06), and awareness of the presence of agencies supporting abuse victims and a hotline for reporting (P = 0.68 and 0.88). Significantly higher odds of reporting were associated with a higher score of professional attitudes towards reporting (AOR = 1.87, P = 0.003) and a lower score of negative perception of workplace commitment to reporting (AOR = 0.77, P = 0.04).

**Table 3:** Factors association with reporting suspected abuse

Factors	AOR (95% CI)	P-value
Received training to manage abuse (yes vs no)	0.77 (0.16, 3.69)	0.74
Number of patients suspected of being abused examined	1.01 (0.99, 1.03)	0.52
Dentist’s age	1.08 (1.00, 1.16)	0.06
Being aware of the presence of agencies protecting the abused (yes vs no)	1.40 (0.28, 7.00)	0.68
Being aware of the presence of a hotline to report suspected abuse (yes vs no)	1.16 (0.16, 8.28)	0.88
Professional attitude score	1.87 (1.23, 2.86)	0.003*
Score for negative perception of workplace commitment to report suspected abuse	0.77 (0.60, 0.99)	0.04*

AOR: adjusted odds ratio, CI: confidence interval

*Statistically significant at P < 0.05

## Discussion

The findings showed that about 43% of the Egyptian dentists in the study reported that they suspected abuse in patients they examined the previous year and 4% of those reported their suspicions. Reporting was significantly associated with more positive professional attitude towards reporting and less negative perception of workplace commitment to reporting which are motivation factors of the COM-B model. There was no significant association between reporting suspected abuse and the capability or opportunity factors of the model. The study hypothesis is, thus, partly supported.

The present study has several strengths including the large number of Egyptian dentists from multiple sectors and areas in Egypt ensuring the greatest representativeness geographically and across sectors. The study also uses a robust theoretical framework to assess factors associated with reporting suspected abuse. The importance of the COM-B model is that it defines these associations in an actionable framework with factors that can be targeted, improved, and customized to various settings by policymakers rather than factors identifying non-modifiable attributes of those who report. This helps improve the preparedness of the healthcare system to support the achievement of Sustainable Development Goal (SDG) #3 by ensuring the health and wellbeing of vulnerable groups such as women and children and SDG #5 by empowering women and preventing violence against them [28]. The study, however, has some limitations including the cross-sectional design which cannot prove causality and the convenience sampling which limits the generalizability of findings. Nevertheless, the study has some important findings.

First, only a minor portion of those who suspected abuse actually reported their suspicions. This level should be interpreted with caution considering the non-statistical nature of the sample. However, this low level agrees with similar levels in Middle Eastern countries like Saudi Arabia [24] and Turkey [25] and is much lower than the levels reported in western countries such as Norway [26], and the UK [27]. These differences may be explained by the definition of abuse in different cultures and countries. For example, the most frequent reason for reporting suspected abuse by Norwegian dentists was skipping a dental appointment and severe caries in children [28]. If visiting a dentist on pain is the norm and systematic dental care with regular check-ups is not available, skipped dental visits and severe caries in children may not be considered to indicate abuse. Thus, a clear definition is needed of what abuse is and it should be developed taking into consideration the principles of preserving human dignity and international guidelines.

Second, the findings showed negative perception of workplace commitment to report suspected abuse and low awareness of whether the law mandates reporting which affect dentists’ motivation to report. Mandated reporting schemes may be regarded as imperfect as they produce many unsubstantiated reports. However, without a system of mandated reporting, a society may be far less able to protect children because many cases of suspected abuse and neglect will not come to the attention of authorities [29]. In Egypt, the law does not mandate reporting of suspected abuse in children and health care professionals are not penalized if they do not report such cases [30]. Reporting, however, is mandated in many countries [5, 31, 32]. Laws which protect dentists who report suspected abuse and, if needed, penalize those who fail to report- may act as incentives promoting reporting [33]. On the other hand, unclear laws that do not explicitly mandate that dentists report suspected abuse may lead to confusion about dentists’ role and lead to subjective organizational or professional interpretation and unstandardized procedures [2]. Also, the perceived inefficiency of the system to protect and support abuse victims may deter dentists from reporting suspected abuse [34]. Concerted efforts are needed to revise the laws and bylaws governing health care professionals’ roles in this respect to ensure clarity and to support efforts to control the problem.

Third, the study showed low awareness of the presence of agencies supporting abuse victims or a hotline for reporting, lack of clarity about whether the law mandates reporting, and inadequate understanding of workplace rules governing reporting. This shows a gap between the existence of policies and laws and their implementation. Interventions based on dissemination and implementation science are needed so that the existing policies can be translated into practices and greater uptake of guidelines is ensured.

Fourth, the non-significant association between reporting suspected abuse and receiving training indicate that more and different training model is needed. Previous research showed that dentists without training to manage suspected abuse were less likely to know how to report suspected abuse than those with training [35]. Also, prior studies including Egyptian physicians [30] and primary health care workers [36] similarly showed that health professionals needed training to be familiar with rules and supporting bodies so there seems to be a consensus that this training gap needs to be filled. Training that focuses on promoting positive attitudes towards reporting and increases awareness of existing laws, policies, and agencies in local context may lead to more reporting than training which aims exclusively at increasing the knowledge of the manifestations of abuse and how to identify abuse victims.

## Conclusion

The study showed that out of 100 Egyptian dentists, 40 would suspect abuse in their patients but less than 1 would report their suspicions. The dentists who reported these suspicions had better professional attitude towards reporting and greater perception of their workplace commitment to support reporting suspected abuse. Current models of training, the mere existence of national agencies supporting abuse victims, and a national hotline for reporting seemed to be not related to actual reporting.

## Data Availability

The dataset analysed in the current study is not publicly available because it is used for other publications at the present time but is available from the corresponding author on reasonable request.

## Abbreviations

AOR: Adjusted odds ratio
CI: Confidence interval
COM-B: Capability, Opportunity, Motivation, Behaviour model
KMO: Kaiser-Meyer-Olkin test
NGO: Non-governmental organisation
PCA: Principal Component Analysis
SD: Standard deviation
UK: United Kingdom
